# Pediatric hospital admissions in Indigenous children: a population-based study in remote Australia

**DOI:** 10.1186/s12887-017-0947-0

**Published:** 2017-11-22

**Authors:** Philippa J. Dossetor, Alexandra L. C. Martiniuk, James P. Fitzpatrick, June Oscar, Maureen Carter, Rochelle Watkins, Elizabeth J. Elliott, Heather E. Jeffery, David Harley

**Affiliations:** 1 Clinical Medical School, College of Medicine, Biology & Environment, Australian National University, 97/2 Edinburgh Ave, Canberra, ACT 2601 Australia; 2 University of Sydney, Discipline of Paediatrics and Child Health, Sydney Medical School, Sydney, Australia; 3 Poche Centre for Indigenous Health, University of Sydney, Sydney, NSW Australia; 4 Dalla Lana School of Public Health, University of Toronto, Toronto, Canada; 5 The George Institute for Global Health, PO Box M201, Missenden Rd, Sydney, 2050 Australia; 6 Population Sciences Division, Telethon Kids Institute, The University of Western Australia, Perth, Australia; 7 Marninwarntikura Women’s Resource Centre, Fitzroy Crossing, Australia; 8 School of Arts and Science, University of Notre Dame, Broome, Australia; 9 Nindilingarri Cultural Health Services, Fitzroy Crossing, Australia; 10 RPA Newborn Care, Royal Prince Alfred Hospital, Sydney, NSW Australia; 11 National Centre for Epidemiology and Population Health, Australian National University, Building 62, Corner of Eggleston and Mills Roads, Canberra, ACT 0200 Australia; 12 Sydney School of Public Health, University of Sydney, Sydney, NSW Australia; 13 The Sydney Children’s Hospital Network (Westmead), Westmead, Australia

**Keywords:** Hospitals, pediatric, Pediatrics, Health services, indigenous, Australia, Child, Rural health services, Oceanic ancestry group, Rural and remote

## Abstract

**Background:**

We analysed hospital admissions of a predominantly Aboriginal cohort of children in the remote Fitzroy Valley in Western Australia during the first 7 years of life.

**Methods:**

All children born between January 1, 2002 and December 31, 2003 and living in the Fitzroy Valley in 2009–2010 were eligible to participate in the Lililwan Project. Of 134 eligible children, 127 (95%) completed Stage 1 (interviews of caregivers and medical record review) in 2011 and comprised our cohort. Lifetime (0–7 years) hospital admission data were available and included the dates, and reasons for admission, and comorbidities. Conditions were coded using ICD-10-AM discharge codes.

**Results:**

Of the 127 children, 95.3% were Indigenous and 52.8% male. There were 314 admissions for 424 conditions in 89 (70.0%) of 127 children. The 89 children admitted had a median of five admissions (range 1–12). Hospitalization rates were similar for both genders (*p* = 0.4). Of the admissions, 108 (38.6%) were for 56 infants aged <12 months (median = 2.5, range = 1–8). Twelve of these admissions were in neonates (aged 0–28 days).

Primary reasons for admission (0–7 years) were infections of the lower respiratory tract (27.4%), gastrointestinal system (22.7%), and upper respiratory tract (11.4%), injury (7.0%), and failure to thrive (5.4%). Comorbidities, particularly upper respiratory tract infections (18.1%), failure to thrive (13.6%), and anaemia (12.7%), were common.

In infancy, primary cause for admission were infections of the lower respiratory tract (40.8%), gastrointestinal (25.9%) and upper respiratory tract (9.3%). Comorbidities included upper respiratory tract infections (33.3%), failure to thrive (18.5%) and anaemia (18.5%).

**Conclusion:**

In the Fitzroy Valley 70.0% of children were hospitalised at least once before age 7 years and over one third of admissions were in infants. Infections were the most common reason for admission in all age groups but comorbidities were common and may contribute to need for admission. Many hospitalizations were feasibly preventable. High admission rates reflect disadvantage, remote location and limited access to primary healthcare and outpatient services. Ongoing public health prevention initiatives including breast feeding, vaccination, healthy diet, hygiene and housing improvements are crucial, as is training of Aboriginal Health Workers to increase services in remote communities.

## Background

There is a health disparity between Indigenous and non-Indigenous Australians, most prominently reflected in the ‘gap’ in life expectancy of 10.6 years for men and 9.5 years for women [1]. The infant mortality rate is 6.1 per 1000 live births for Indigenous infants versus 3.4 for non-Indigenous Australian infants (< 12 months) [2]. There are statistically significant (*p* < 0.05) differences in child mortality rates between Indigenous and non-Indigenous children with rate ratios of 2.1 (M) and 3.0 (F) for mortality in children aged 1–4 and 2.4 (M) and 2.3 (F) for children aged 5–14 years. [3]. In 2014 the under-five mortality rate in Australia was 4.0 per 1000 live births [4]. During 2008–10, the infant mortality rate for children living in remote and very remote areas was double that of infants living in major cities (6.8 versus 3.9 per 1000 live births); and for children aged 1–14 years the mortality rate in remote and very remote areas was three times higher (31.0 versus 11.0 per 100,000 children) [5]. Remote indigenous families face challenges when seeking health care due to cultural factors, historically poor interactions with health care systems, and a lack of cultural awareness and sensitivity of non-Indigenous health workers. Additional challenges include geographical isolation, lack of transport and environmental and climatic factors [5], and limited access to specialist pediatric services, all of which are compounded by language barriers [6, 7]. Health outcomes to an extent reflect health service effectiveness [8]. The Australian Government has committed to ‘Closing the Gap’ and removing the health inequity experienced by Australia’s Indigenous people.

**Fig. 1:**
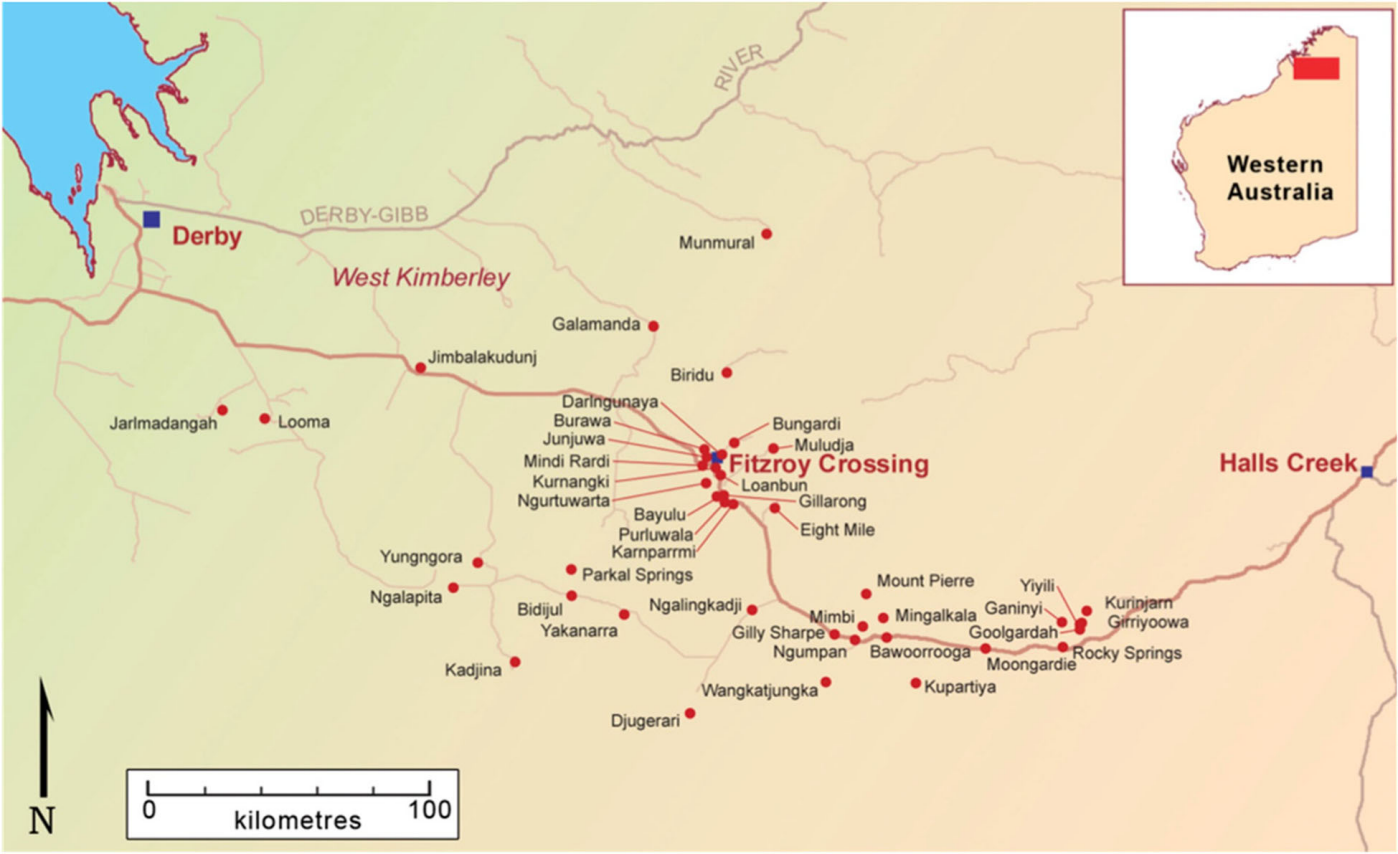
Fitzroy Crossing and surrounding communities, reproduced with permission from Frances Morphy, 2009 [9]

The Fitzroy Valley is an area of 2500 km^2^ in the Kimberley region of Western Australia (WA), 400 km east of Broome [9]. There are 45 communities within a 200 km radius of Fitzroy Crossing town, and the town and surrounding communities are all classified as ‘very remote’ [9] (see Fig. 1). Approximately 4500 people live in the Fitzroy Valley and the majority (93%) are Aboriginal and comprise five distinct language groups (Bunuba, Walmajarri, Wangkatjungka, Gooniyandi and Nyikina peoples) [9]. Some communities are only accessible by dirt road and access is restricted for weeks by annual flooding. Communities are located up to 190 km (3.5 h’ drive) from the district hospital in Fitzroy Crossing. Other hospitals are located in Derby (259 km), Halls Creek (289 km), Broome (397 km) and Princess Margaret Hospital in Perth (2566 km) [10]. In the Fitzroy Valley many of the aforementioned barriers for access to health care exist. In addition, it is difficult to attract and retain health professionals because of the remote location, professional isolation and lack of accommodation.

In 2009, female Indigenous leaders from Fitzroy Crossing invited researchers and clinicians from the University of Sydney Medical School (Discipline of Paediatrics and Child Health, and the George Institute for Global Health) to help them advance a strategy they had developed to address Fetal Alcohol Spectrum Disorders (FASD) called The Marulu Strategy [11]. A partnership was formed with Nindilingarri Cultural Health Services and the Marninwarntikura Women’s Resource Centre in Fitzroy Crossing. The research groups agreed to conduct a population-based study (The Lililwan Project), to determine the prevalence of FASD [12]. Lililwan is a word in the Kimberley Kriol language, which means ‘all the little ones.’

Families of children born in 2002 and 2003 and living throughout the Fitzroy Valley were invited to participate in the Lililwan Project. In Stage 1 (2010) parents and caregivers were interviewed and maternal and child medical records reviewed for information on health problems and hospitalizations in the first 7 years of life. In 2011, children (aged 7–9 years) underwent comprehensive health and development assessments by a multidisciplinary team and individual management plans were developed [12].

Our primary aim was to describe the frequency, primary reasons for, and comorbidities at hospital admission for a very remote dwelling population of primary school-aged children. Secondary aims were to test the hypotheses that: 1. Indigenous children have more admissions than non-indigenous children; 2. Alcohol exposure *in utero* adversely affects child health; 3. Infections are the most common cause of admission; and 4. Poor social determinants increase admission frequency.

## Methods

### Identification of the Lililwan cohort

**Fig. 2:**
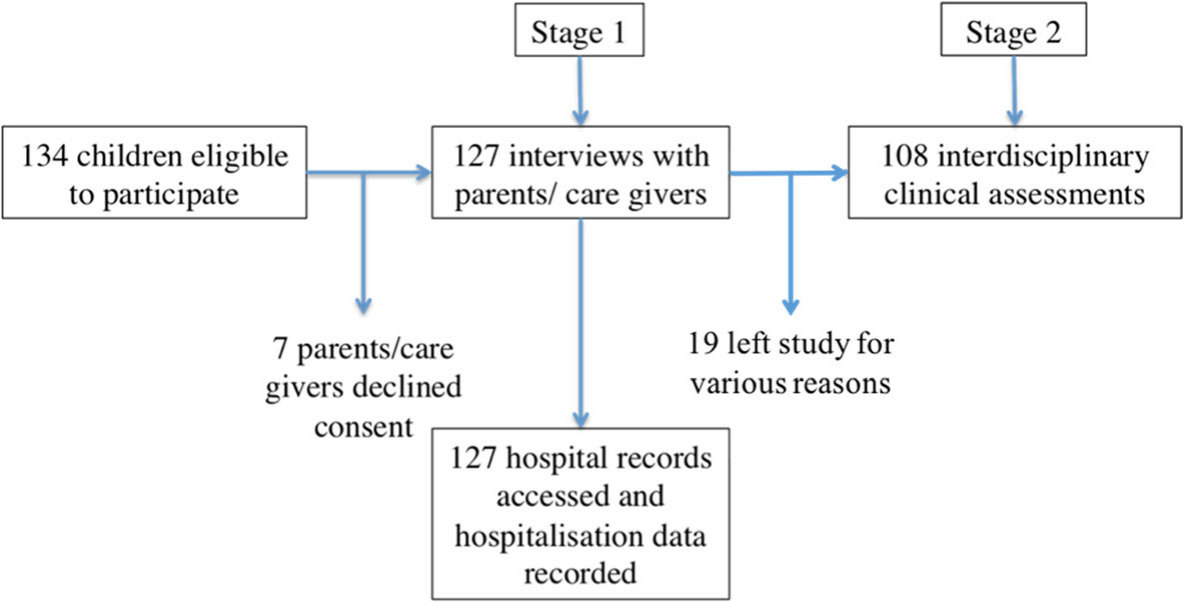
Participatory flowchart of a population-based cohort of children (b.2002/3), living in Fitzroy Valley in 2010–11

The Lililwan Project is a population-based study of FASD prevalence, using active case ascertainment and methods have been published [12–15]. Children born between January 1st 2002 and December 31st 2003 who were living in the Fitzroy Valley during 2010–11 were identified using the Fitzroy Valley Population Project and Communicare™ databases (*n* = 134). Consent for participation was obtained from a parent or caregiver for 127 (95%) of children and there were no exclusion criteria (Fig. 2) [12].

### Stage 1

The cohort was characterised through structured interviews with parents or caregivers using a reliable tool developed in consultation with the local Aboriginal community [14]. Health professionals conducted interviews alongside Community Navigators and both underwent training in use of the questionnaire [14]. Data were collected on prenatal exposures, health, living conditions and schooling [12]. Parents were asked about hardships including food insecurity (times during the childhood when the parent had worried about not having enough food) and financial concerns (reports of adults not having enough money) [12]. Birth weights were plotted on WHO Child Growth Standard sex and age-appropriate centile charts to derive the birth centiles [16, 17].

### Hospitalization data

For each child, admissions to Fitzroy Crossing Hospital were recorded from birth till age seven. We retrospectively searched hospital records till 2011 using the Communicare™ electronic health and practice management system and cross-checked against hard copy case note files. Data extracted included lifetime hospital admissions; date, reasons for, and comorbidities at admission; birth and growth parameters; and medical diagnoses.

### Stage 2: FASD assessments

An interdisciplinary team conducted diagnostic assessments for FASD during 2011 [15] using the Canadian FASD diagnostic guidelines [18]. We also documented current health problems. FASD is caused by exposure to alcohol *in utero* and includes Fetal Alcohol Syndrome (FAS), partial FAS (pFAS) and Neurodevelopmental Disorder associated with Alcohol Exposure (ND-AE).

### Coding of hospitalization datasets

Information from interviews and record reviews was entered into a Microsoft Access database, checked and coded. When children presented to the hospital with multiple conditions, the primary diagnosis was determined by a senior pediatrician and additional co-morbidities recorded. If information was missing or there were discrepancies, we returned to the original patient documentation to clarify diagnoses. When the full date of admission was not recorded, the first date of the stated month or year was documented. Diagnoses assigned at discharge were coded using the International Statistical Classification of Diseases and Related Health Problems, Tenth Edition, Australian Modification (ICD-10-online, World Health Organization (WHO)) [19]. Each diagnosis was categorised by a main, sub and specific code which ranged from three to seven characters including a letter and at least two numeric codes. Missing data were coded as 999. We used the most accurate code possible for each diagnosis.

### Statistical analysis

Data were analysed using IBM SPSS Statistics for Mac (version 22.0.0.0 Armonk, NY, USA). Descriptive analysis was performed to obtain frequencies, medians and prevalence estimates. Chi-squared tests were used to examine associations between dichotomous variables. Although participation rates for this study were excellent (127/134), the cohort was small and continuity corrected values were reported where necessary for Chi-squared values. Independent sample t-tests were used for continuous data.

### Ethics approval and consent

Ethics approval for the Lililwan Project was obtained from the University of Sydney Human Research Ethics Committee (Lililwan Project Approval numbers 12527, 13187, 13551), the Western Australian Aboriginal Health Ethics Committee (Approval numbers 271–01/10, 319–10/10, 344–04/11), the WA Country Health Service Human Research Ethics Committee (Approval numbers 2010:01, 2010:28, 2011:04), and the Kimberley Aboriginal Health Planning Forum Research Sub-committee (Approval numbers 2010–001, 2010–001, 2010–001). The Health Services project was an extension of the Lililwan Project and was approved by the Western Australian Aboriginal Health Ethics Committee (Approval number 344–04/2011) and the WA Country Health Service Human Research Ethics Committee (Approval number 2013:18).

Consent to participate was obtained for each child (7–9 years) from all parents or guardians through ‘Community Navigators’ (Aboriginal members of the research team with local language skills and knowledge of cultural protocols) who explained the purpose and nature of the study in local language of the parent’s preference. Information statements and consent forms were additionally provided to families, or read to them, in the local Aboriginal language of their choice, Kimberley Kriol or English.

## Results

### Characteristics of the Lililwan cohort

This descriptive study includes a unique, predominantly Indigenous (95.3%), population of primary school aged children, who live in a very remote part of Australia and have limited access to health care services. Detailed characteristics of the Lililwan cohort have been published [18]. This study had a 95% participation rate for Stage 1, which included consent for the documentation of hospital admissions data (Fig. 2). The cohort is 52.8% male (Table 1). All children lived in very remote communities. Nearly half (45.7%) live in sub-regional hub communities (population 200 < 999) or satellite communities (population < 200).

**Table 1:** Characteristics of the Lililwan cohort

Variable	Total (*n* = 127) n (%)	Hospital Admissions (*n* = 89)	No Hospital Admissions (*n* = 38)	*P*-value (Chi^2^ Test)
Indigenous (child)	121 (95.3)	88 (98.9)	33 (86.8)	0.009*
Sex male	67 (52.8)	49 (55.1)	18 (47.4)	
Place of residence at time of assessment (*n* = 127)				
Very Remote (ARIA+)^d^	127 (100.0)			
a) Town (population 1000–9999)	43 (33.9)	26 (29.2)	17 (44.7)	
b) Outer suburbs (within 30 km of remote town)	26 (20.5)	17 (19.1)	9 (23.7)	
c) Sub-regional hub (population 200–999)	31 (24.4)	22 (24.7)	9 (23.7)	
d) Satellite community (population < 200)	27 (21.3)	24 (27.0)	3 (7.9)	
a + b) Town or Outer suburbs	69 (54.3)	43 (33.9)	26 (68.4)	0.037*^e^
c + d) Hub or satellite community (*n* = 127)	58 (45.7)	46 (51.7)	12 (31.6)	
Food insecurity (Y/N) (*n* = 124)	52 (41.9)	43 (48.3)^e^	9 (24.3)^#^	0.01*
Financial concerns (*n* = 124)	47 (37.9)	37 (42.0)^e^	10 (27.0)^#^	
Number living in overcrowded households *n* = 124	42 (33.1)	33 (37.0)	9 (23.7)	
Number in household - median (range)^#^	4 (2–16)	4 (2–12)	4 (2–16)	
Number of older siblings – median (range)^#^	2.0 (0–7)	2.0 (0–7)	2.0 (0–3)	
Number of younger siblings – median (range)^#^	1.0 (0–3)	1.0 (0–3)	1.0 (0–3)	
Mothers age at pregnancy (median, range)^#^	23 (14–43)	23 (15–36)	24 (14–43)	
Gestation (*n* = 118)				
≥ 37 weeks (term)^#^	103 (87.1)	75 (84.3)	28 (73.7)	
< 37 weeks (preterm)	15 (12.7)	11 (12.4)	4 (10.5)	
< 28 weeks (extremely preterm)	4 (3.4)	3 (3.5)	1 (2.6)	
Alcohol exposed in utero (Y/N) (*n* = 122)	67 (52.8)	50 (56.2)^#^	17 (44.7)^#^	
FASD diagnosis (*n* = 108)	21 (19.4)	19 (21.3)	2 (5.7)^#^	
Microcephaly at assessment n=108^a^	16 (14.8)	12 (13.5)	4 (10.5)	
Low Birth Weight (*n* = 105)¶				
Very low birth weight (< 1500 g)	3 (2.9)			
Low birth weight (< 2500 g)	18 (17.1)			
Birth weight ≤ 3^rd^ percentile^b^	2 (1.9)			
Birth weight ≤ 10^th^ percentile^b^	11 (10.5)			
Growth deficiency at any age (Y/N) (*n* = 92)^b^	32 (34.8)	26 (41.3)	6 (20.6)	
Any hearing loss (*n* = 98)^c^	54 (55.1)	40 (57.1)	14 (50)^#^	
Exposure to cigarettes (nicotine) in utero (Y/N) *n* = 119	76 (65.5)	53 (63.1)^#^	23 (65.7)^#^	
Marijuana exposure in utero (Y/N) *n* = 119	16 (13.4)	12 (14.3)^#^	4 (11.4)^#^	
Both cigarettes and marijuana (Y/N) *n* = 119	16 (13.4)	12 (14.3)^#^	4 (11.4)^#^	

#indicates missing values as data due to unavailable data

¶ Data unavailable for hospitalised and non-hospitalised sub-groups

*indicates significant difference *p* < 0.05

^a^Microcephaly: head circumference < 3rd percentile using WHO Child Growth Standards

^b^Growth deficiency at any age: height or weight < 10th centile recorded at any age from birth until time of assessment in Stage 2 (7 to 9 years of age)

^c^Hearing loss: Determined by an audiologist who conducted tympanometry, audiometry, video-otoscopy and Listening in Spatialized Noise - Sentences Test (LiSN-S) for Central Auditory Processing Disorder assessments

^d^Remoteness was classified using the Australian Statistical Geography Standard (ASGC) Accessibility/Remoteness Index of Australia (ARIA+), developed by the National Centre for the Social Applications of Geographic Information Systems (GISCA) and the Commonwealth Department of Health and Aged Care (DH&AC) in order to classify remoteness of 12,000 populations in Australia based on physical road distance measurements to nearest service centres

^e^Note significance here was calculated for a + b versus c + d

The median age of mothers at their child’s birth was 23 (range 14–43) and 87.1% (*n* = 118) of pregnancies continued to term (≥ 37 weeks gestation). Fifteen (12.7%) children were born preterm (< 37 weeks) and four children (3.4%) were extremely preterm (< 28 weeks) (Table 1). Of the children, 17.1% had low birth weight (< 2500 g) and 2.9% very low birth weight (< 1500 g) [16, 17].

The median number of residents per house was four (range 2–16) and a third (33.1%) of the cohort lived in households considered overcrowded by the parents/caregivers. In 2011 the median number of older siblings was two (range 0–7) and the median number of younger siblings one (range 0–3). Exposure to maternal alcohol (52.8%) and cigarette (65.5%) use in pregnancy was common, and 13.4% of the cohort was exposed to both marijuana and tobacco prenatally.

During the child’s first 7 years 37.9% (*n* = 124) of caregivers reported financial concerns and 41.9% (*n* = 124) reported food insecurity.

### Hospitalizations

Of the 127 children, 89 (70%) were admitted to hospital during their first 7 years of life (Table 2). There was a total of 314 admissions for 424 reasons, with a median of 2.0 admissions per child (range 0–12) or 5.0 (range 1–12) per admitted child. There were 108 admissions for 56 children in infancy (the first year of life). The median number of admissions for these 56 children was 2.5 (range 1–8). Males and females had a similar number of admissions both before 7 years of age and during infancy (Table 2). Additional analyses were performed to examine children who had multiple admissions by comparing children who were admitted 0 to 2 times with children admitted 3 to 12 times however no significant differences were found.

**Table 2:** Lifetime (0–7 years) hospital admissions for 127 children born 2002/2003 living in Fitzroy Valley

Hospital Admissions	Children assessed Median (Range)
Childhood (0–7 years)	
Number of children with one or more admissions by 7 years (*n* = 127)	89 (70.0%)
Total number of hospital admission (number of reasons for admission)	314 (424)
Median number of admissions (range) per child by 7 years	2.0 (0–12)
Median number of admissions (range) per admitted child by 7 years (*n* = 89)	5.0 (1–12)
Number of admissions by sex	
Male median (Interquartile range)	3.0 (4)
Female median (Interquartile range)	2.0 (3)
Infancy (0–1 years)	
Number of infants with one of more admissions by 1 year (*n* = 127)	56 (44.1%)
Total number of admissions in infancy (number of reasons for admission)	108 (135)
Median number of admissions (range) (*n* = 127)	0 (0–8)
Median number of admissions (range) per admitted infant (*n* = 56)	2.5 (1–8)
Number of admissions by sex for infants	
Male median (Interquartile range)	3.0 (5)
Female median (Interquartile range)	2.0 (4)
Admissions by prenatal alcohol exposure (*n* = 122)^a^	
Prenatal alcohol exposure (*n* = 67 children) median (Interquartile range)	2.0 (4)
No prenatal alcohol exposure (*n* = 55 children) median (Interquartile range)	1.0 (3)
Admissions for children with FASD (*n* = 108)^a^	
Children with FASD (*n* = 21 children) median (Interquartile range)	3.0 (4)
Children without FASD (*n* = 87 children) median (Interquartile range)	2.5 (3)

^a^Number of children that had information available on alcohol exposure *in utero*

**Fig. 3:**
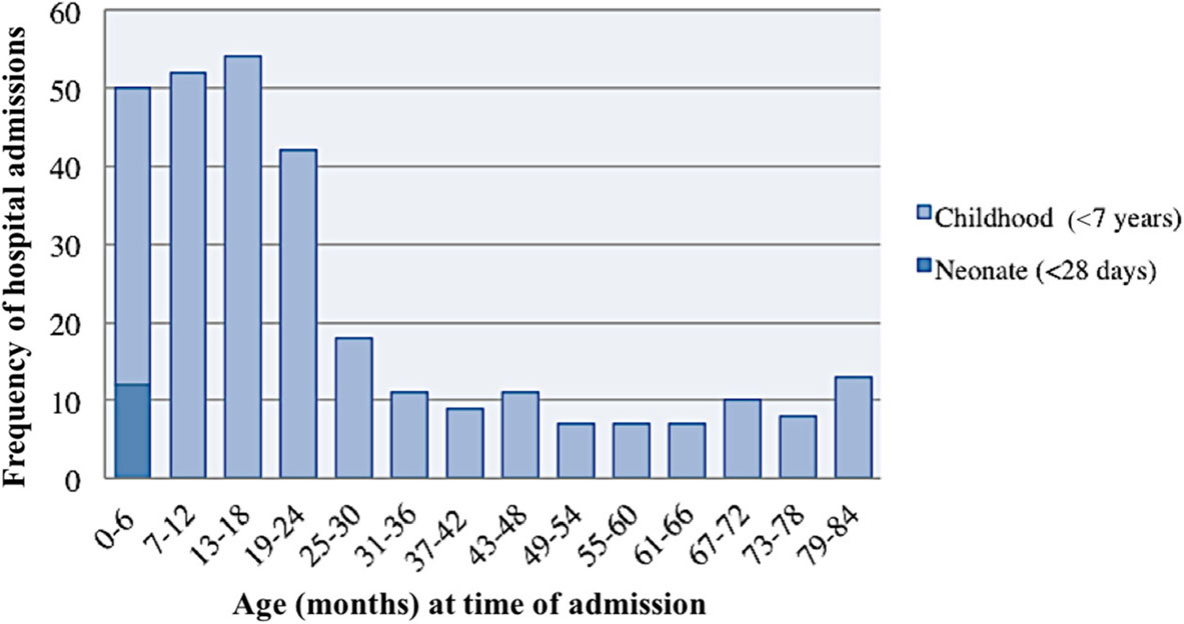
Frequency and age of admissions in children (0–7 years) and neonates (<28 days)

Children living in sub-regional hub (population 200 < 999) or satellite community settings (population < 200) had a higher rate of admission than children living in outer suburbs (within 30 km of a remote town) or a town (population 1000–9999) (*p* = 0.037) (Table 1). A large proportion of admissions (63.0%) occurred before the age of 2 years, one third (34.4%) in infancy, and 3.8% in the neonatal period (<28 days) (Fig. 3). Most initial admissions occurred in the first 2 years of life and there was no association with sex (Table 2).

Food insecurity was reported more commonly in children who were hospitalised (48.3%) than those who were not (23.7%), *p* = 0.01 (Table 1). None of the variables: gestational age; mother’s age at pregnancy; the number living in the household; financial concerns; or microcephaly were significantly associated with hospital admissions.

**Fig. 4:**
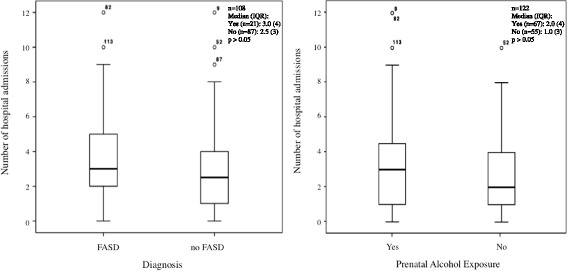
Admissions in FASD (*n* = 21) and non-FASD (*n* = 87) children, and with (*n* = 67) and without PAE (*n* = 55)

For children in the Lililwan cohort who completed Stage 1 (*n* = 127) [16] and for whom we had data on alcohol exposure *in utero* (*n* = 122), there was no difference in the number of admissions between those exposed (*n* = 67; median 2.0, range 0–12) and those not exposed to alcohol (*n* = 55; median 1.0, range: 0–10) (Table 1, Fig. 4).

For participants who completed Stage 2 (*n* = 108) there was no difference between the number of admissions in children with (*n* = 67) and without (*n* = 55) prenatal alcohol exposure (PAE) and with (*n* = 21) and without FASD (*n* = 87) (Fig. 4). A majority (90%) of children with FASD had been admitted to hospital at least once.

### Reasons for hospital admissions

Infection was the most frequent primary reason for admission (79%) in children aged under 7 years. Infections of the lower (27.4%) and upper (11.4%) respiratory tract, the gastrointestinal system (22.7%) and skin (5.6%) were most common. Injury (7%) and failure to thrive (5.4%) were also common primary reasons for admission (Table 3). Common comorbidities at the time of admission included upper respiratory tract infections (URTI) (18.1%), failure to thrive (13.6%), anaemia (12.7%), gastroenteritis (7.3%), asthma (3.6%) and unspecified fever (3.6%) (Table 3).

**Table 3:** Predominant primary reasons for admission and common comorbidities at presentation in childhood (0–7 years) (*n* = 89)

	Conditions (ICD Code)	Frequency of admissions	Percentage of total
Top Primary Reasons for Presentation			
Lower respiratory tract infection		86	27.4
	Lower respiratory tract infection (J22)	74	
	Pneumonia (J18.9)	10	
	Bronchiolitis (J21.9)	2	
Gastroenteritis		71	22.7
	Infectious gastroenteritis (A09.9)	68	
	Vomiting (R11)	3	
Upper respiratory tract infection		36	11.4
	Otitis media (H66.3, H66.9, H70.019, H73.1)	15	
	Upper respiratory tract infection (J06.9)	14	
	Croup (J05.0)	4	
	Tonsillitis (J03.9, J35)	1	
	Pharyngitis (J02.9)	1	
	Epiglottitis (J05.1)	1	
Injury	Traumatic brain injury (S06.01), head injury (S09.9), fractured femur (S27.0), dislocated elbow (S53.10), finger injury (S69.7), soft tissue injury, hip (S76.7), superficial injury of leg/ft (S80.84, S90.82, S91.3), traumatic amputation of leg (T13.6), animal bite (dog or snake) (T14.1), foreign body (T16), corrosive burn (T24.0, T30.0), poison ingestion (T38.3)	23	7.0
Skin infection		18	5.6
	Impetigo (L01.0)	1	
	Cutaneous abscess, furuncle, carbuncle (L02.0–.9)	7	
	Cellulitis (L03.9)	6	
	Skin sore (L98.9)	3	
	Lymphadenitis (L04.3)	1	
Failure to thrive	(R62.8)	17	5.4
Genitourinary		14	4.4
	Urinary tract infection (N39.0)	12	
	Post infectious complications (N05.9, N12)	2	
Asthma	(J45.9)	10	3.2
Subtotal admission reasons		275	87.1
Other		39	12.9
Total Admission reasons		314	100
Top Comorbidities at Presentation			
Upper respiratory tract infection	H66.3, H66.9, J03.9, J05.0, J06.9	20	18.1
Failure to thrive	R62.8	15	13.6
Anaemia	D64.9	14	12.7
Gastroenteritis	A07.1, A08.0, A09.9, E87.2 (acidosis)	8	7.3
Asthma	J45.9	4	3.6
Fever, unspecified	R50.9	4	3.6
Subtotal comorbidities		65	58.9
Other		45	41.1
Total comorbidities		110	100

One child was admitted for meningococcal meningitis and another for acute rheumatic fever. Two were admitted with mitral regurgitation secondary to rheumatic heart disease. Two cases of post-infectious genitourinary complications were recorded (glomerulonephritis and tubulo-interstitial nephritis).

In infancy (< 1 year), infection was the predominant primary reason for admission (90%) (Table 4). The most common infections in infancy were lower respiratory tract infections (LRTI) (40.8%) and gastroenteritis (25.9%). Admissions for injury were also recorded (3.6%). Comorbidities included URTI (33.3%), failure to thrive (18.5%) and anaemia (18.5%). Injuries included traumatic brain injury; snake or dog bites; or falls resulting in head injuries, which often required sutures.

**Table 4:** Predominant primary reasons for admission and common comorbidities at admission in infancy (0–1 years) (*n* = 56)

	ICD Codes/Conditions	Frequency of Admissions	Percentage of Total Admissions
Top Reasons for Primary Admission (<1 YEAR)			
Lower respiratory tract infection		44	40.8
	Lower respiratory tract infection (J22)	42	
	Bronchiolitis (J21.9)	2	
Gastroenteritis		28	25.9
	Infectious gastroenteritis (A09.9)	27	
	Vomiting (R11)	1	
Upper respiratory tract infection		10	9.3
	Otitis media (H66.3, H66.9)	5	
	Upper respiratory tract infection (J06.9)	4	
	Croup (J05.0)	1	
Genitourinary	Urinary Tract Infection (N39.0)	5	4.6
Injury	Traumatic brain injury (S06.01), head injury (S09.9), fractured femur (S72.9), dog or snake bite (T14.1)	4	3.6
Subtotal admission reason		92	84.2
Other		16	15.8
Total Primary admission reason		108	100
Top Comorbidities at Admission			
Upper respiratory tract infection	H66.3, H66.9, J06.9	9	33.3
Failure to thrive	R62.8	5	18.5
Anaemia	D64.9	5	18.5
Subtotal comorbidities		19	70.3
Other		8	29.7
Total comorbidities		27	100

## Discussion

Over two thirds (70%) of the remote Fitzroy Valley child cohort were admitted to hospital at least once during early childhood (aged 0–7 years), and over one third were admitted during infancy, with 12 admissions (4%) in the neonatal period. The proportion of males and females admitted was similar. The predominant primary reason for admission was infection (79%) including infection of the lower and upper respiratory tract; gastrointestinal system; skin; and urinary tract. Common comorbidities in childhood and infancy included URTIs (including otitis media), anaemia and failure to thrive.

Children were admitted up to 12 times in the first 7 years of life, and up to eight times in the first year of life. The number of neonatal admissions may be lower than expected, considering the high proportion (17.1%) of new-borns with low birth weight. Such children often have had a prolonged duration of hospitalization after birth and therefore rates of neonatal admissions may be under-represented, particularly in remote areas where distances to hospital are greater, [20]. In addition, care seeking is likely less common in remote than metropolitan areas where health care facilities are readily accessible [21].

Families living in the remote Fitzroy Valley region experience multiple barriers to accessing health care services including lack of transport, lack of infrastructure, long distances, and environmental factors resulting in some communities being cut off from road access during the wet season [8, 22]. If children live considerable distances from a hospital they are likely to present late with more severe illness. Conversely, children from remote areas who present to hospital with less severe illness may be admitted because the remoteness of their homes renders adequate follow up difficult [8, 22]. This concept is explored for pregnant women in the Three Delays Model in low- and middle-income countries in which there are three potential levels of barriers to health care which could also be applied to our cohort: a mother’s decision to seek health care; reaching health facilities; and receiving adequate treatment [7, 23].

Our findings are consistent with national data regarding hospital separations in Indigenous Australians, in remote locations and for male children. In Australia in 2013, in the 0–14 age group, hospital separations were more frequent in Indigenous (10%) than non-Indigenous children (6%) [24]. Hospital separation rates were 551 per 1000 persons for very remote settings and 251 for major cities; and boys aged under 14 years are more likely to be admitted to hospital than girls of the same age [24].

Low-moderate PAE is associated with low birth weight (< 2500 g) [25, 26] and birth weight is a key indicator of health status [27]. Of 127 children in the Lililwan cohort, 52.8% were exposed to alcohol *in utero* and 17.1% were low birth weight. This is much higher than low birth weight rates nationally (12.6% for Indigenous and 6.0% for non-Indigenous births in 2011 [28]) and for WA (6.1% in 2012) [29]. Programs to reduce the prevalence of maternal risk factors, particularly alcohol use, smoking and under-nutrition would increase fetal and neonatal weight. In addition, rural and remote residence has been associated with placental inflammation which is perpetuated by anaemia, genitourinary infections and smoking [30]. Microcephaly was common in our cohort (14.8%) and is likely related to reflect high rates of PAE and FASD [31].

Many of the conditions that precipitated admissions in our cohort were feasibly preventable. The Australian Institute of Health and Welfare estimates that for Indigenous Australians the overall rate of potentially preventable hospitalizations is greater than three times the rate in non-Indigenous Australians [24]. Globally, a large proportion (36%) of deaths in children aged under 5 years result from LRTI and gastroenteritis, and are preventable through breast feeding, vaccination, nutrition, sanitation and clean water programs [32–34]. In 2014 globally, LRTI ranked second, and diarrhoeal disease was fifth for Years of Life Lost (YLL). However, due to interventions there was a 30% global decrease in YLL from LRTI and a 40% global decrease from diarrhoeal diseases between 2000 and 2012 [35]. LRTI and gastroenteritis are also high contributors to the burden of ill health in remote Australian Indigenous children and are typical of the burden of disease in children in low- and middle-income countries.

Gastroenteritis was one of the most common reasons for admission in both childhood (0–7 years) and infancy (<1 year) in our study. Admissions attributable to vaccine preventable diseases are 6 times higher in Indigenous than non-Indigenous Australians [24]. *Rotavirus* is the most common cause of gastroenteritis in children globally [36]. The National Rotavirus Immunisation Program only became universal in WA in 2007 [37]. The cohort we studied was born 2002–2003 before Rotavirus vaccine became available and it is likely that the vaccine will substantially decrease rates of gastroenteritis requiring hospitalization among children born after 2007. It was estimated that in 2014 over 90% of children living in WA had full immunisation coverage [37].

Free vaccinations for *Haemophilus influenzae* type B, *Streptococcus pneumoniae* (7vPCV and 23vPPV), and *Bordetella pertussis* (from the Pertussis component of the Diptheria, Tetanus and Pertussis (DTPa-IPV) vaccine) were made available to Indigenous children in WA in 2000, 2001 and 2005 respectively [37]. Immunization records and coverage data were not available for the Lililwan cohort (born in 2002–3), however with good immunization coverage we would expect to see falls in the rates of URTI and LRTI requiring hospital admission [37]. Influenza is a common infection in childhood, but vaccination did not become available to Aboriginal children in WA until 2008 [37]. Rare infections experienced by some children in our cohort, such as meningococcal meningitis, are also vaccine preventable [37].

Some LRTI and gastroenteritis cannot be prevented with vaccines. Similarly, some conditions detected in our cohort, for example failure to thrive and anaemia, have multiple causes including infections, low birth weight, overcrowding, inadequate hygiene, and poor nutrition [5, 8, 10, 38]. In our study, children living with food insecurity had a significantly higher admission rate. Thus, additional disease prevention strategies include early and exclusive breastfeeding during infancy, skin-to-skin kangaroo mother care (KMC) for preterm infants, improvements in nutrition, zinc supplementation, improved hygiene, decreased exposure to cigarette smoke, improved sanitation, clean water supply (for diarrhoea and skin conditions), use of oral rehydration therapy and decreasing indoor air pollution (for pneumonia) [34]. Both breastfeeding [33] and KMC [39] provide economically positive and low cost methods to reduce hospitalizations.

The AIHW reports that injuries requiring hospital admission are more than twice as common in Indigenous than other Australians (41 versus 20 per 1000 admissions, respectively) [24]. Many of the injuries documented in our cohort – snake and dog bites, corrosive burns, traumatic amputation following a motor vehicle accident, falls, poison ingestion, dislocations, head injury and fractures could potentially have been prevented by better supervision of young children, better road and motor vehicle standards, child proof containers for toxins, and other strategies. Data limitations in our study prevented us from understanding fully the circumstances for injuries. Preventive strategies to lessen Indigenous disempowerment and reduce social and family dysfunction may also have a role.

Previous studies examining presentations to a primary health care facility in a remote dwelling Aboriginal child cohort in the NT similarly documented high rates of LRTI, URTI and gastroenteritis [21]. In addition, a high prevalence of scabies and tinea was reported, as were rare conditions including acute post-streptococcal glomerulonephritis and acute rheumatic fever [21]. In our cohort, adequate treatment of Group A *Streptococcus* throat and skin infections could have prevented both rheumatic fever and its complications, including cardiac valvular disease, and post-streptococcal glomerulonephritis.

A Northern Territory study of community clinic presentations reported higher rates of skin infections and scabies than we measured during hospital admissions [21], however this may be due to high frequency and consequent under-reporting. Furthermore, only severe skin infections require hospitalization [21]. Preventative strategies include clean water and hygiene programs.

This project was community led and all interactions between researchers and locals were broached by local Aboriginal language speakers (Community Navigators). The project was population-based so every eligible child was invited to participate and a process of active case ascertainment was used. Although the Lililwan cohort was dispersed across the wide geographic area of the Fitzroy Valley, community leadership resulted in a high consent and participation rate (95%) for Stage 1, which included the hospitalization data.

The questionnaire used in this study was reliable and culturally appropriate, and we used internationally accepted criteria to diagnose FASD [14, 40]. Additionally, a validated instrument was employed to obtain information about alcohol use in pregnancy (AUDIT-Scoring) [15].

There are few studies like ours that describe the lifetime hospitalization of children in remote Australia. We used the only available dataset on hospital admissions but do not have records on length of hospital stay. Each episode of hospitalization was classified according to the primary diagnosis and comorbidities at admission. The reasons for admissions were subsequently coded using the International Classification of Diseases (ICD-10-online), for international comparability. A limitation of the data was the restricted diagnostic laboratory capacity at remote hospitals. Pathological confirmation of diagnoses would have enriched our data, however limited testing is done at Fitzroy Crossing Hospital. Also, this dataset only provides information on children admitted to Fitzroy Crossing hospital, not on children who were transferred and admitted directly to other hospitals in Western Australia (Broome, Derby, Halls Creek or Perth) or Northern Territory (Darwin) and thus may underestimate the true rates of hospitalization.

Nevertheless, our study provides a unique snapshot of the lifetime hospitalizations and needs of children in 45 very remote communities in WA. We acknowledge that the small sample size is a limitation of this study. However, it is a population-based sample in which we have included over 95% of all eligible children in two entire birth cohorts. These data, although not generalizable to all of Australia are likely to be representative of other remote Aboriginal communities in WA, NT, SA, Northern QLD and Western NSW. In these areas there are similar challenges concerning health needs, limited access to health services (transport, geography, climate, infrastructure), and difficulties with attracting and retaining trained health professionals. Rates of hospital admission do not directly reflect the full burden of disease because some conditions prevalent in the community do not require admission [8].

## Conclusion

There is a significant amount of published research about Indigenous health in Australia, particularly at national and state levels. However, community level studies that focus on the morbidity of Indigenous populations, particularly children, are rare, as are studies that describe the utilisation of health services.

We know that Indigenous children in remote settings suffer a range of diseases and infections that are rare in other Australian children [21]. Indigenous children in remote settings often have a complex range of health and developmental issues. Indigenous child health service needs and the context in which services are delivered may thus be radically different from those required in other settings.

Our findings, illustrate the high burden of disease in Aboriginal children living in remote communities and have implications for clinical service delivery, national Indigenous policy, and prevention. We have documented the nature and high rate of hospitalizations in very remote dwelling Indigenous children. The challenge is to design and deliver clinical and public health services and social policies to manage and ameliorate the health burden for Aboriginal children in remote communities. While high quality clinical services remain crucial, high priority should be given to social determinants of health.

## Abbreviations

AIHW: Australian Institute for Health and Welfare
CNS: Central nervous system
FAS: Fetal Alcohol Syndrome
FASD: Fetal Alcohol Spectrum Disorders
IQR: Inter-quartile range
LRTI: Lower respiratory tract infection
NSW: New South Wales
NT: Northern Territory
PAE: Prenatal Alcohol Exposure
pFAS: Partial Fetal Alcohol Syndrome
QLD: Queensland
SA: South Australia
URTI: Upper respiratory tract infection
WA: Western Australia
WHO: World Health Organisation
YLL: Years of Life Lost
